# Diversity and specificity in the interaction between *Caenorhabditis elegans *and the pathogen *Serratia marcescens*

**DOI:** 10.1186/1471-2148-4-49

**Published:** 2004-11-22

**Authors:** Hinrich Schulenburg, Jonathan J Ewbank

**Affiliations:** 1 Department of Evolutionary Biology, Institute for Animal Evolution and Ecology, Westphalian Wilhelms-University, Hüfferstr. 1, 48149 Münster, Germany; 2 Centre d'Immunologie de Marseille Luminy, INSERM/CNRS/Université de la Méditerranée, Case 906, 13288 Marseille Cedex 9, France

## Abstract

**Background:**

Co-evolutionary arms races between parasites and hosts are considered to be of immense importance in the evolution of living organisms, potentially leading to highly dynamic life-history changes. The outcome of such arms races is in many cases thought to be determined by frequency dependent selection, which relies on genetic variation in host susceptibility and parasite virulence, and also genotype-specific interactions between host and parasite. Empirical evidence for these two prerequisites is scarce, however, especially for invertebrate hosts. We addressed this topic by analysing the interaction between natural isolates of the soil nematode *Caenorhabditis elegans *and the pathogenic soil bacterium *Serratia marcescens*.

**Results:**

Our analysis reveals the presence of i) significant variation in host susceptibility, ii) significant variation in pathogen virulence, and iii) significant strain- and genotype-specific interactions between the two species.

**Conclusions:**

The results obtained support the previous notion that highly specific interactions between parasites and animal hosts are generally widespread. At least for *C. elegans*, the high specificity is observed among isolates from the same population, such that it may provide a basis for and/or represent the outcome of co-evolutionary adaptations under natural conditions. Since both *C. elegans *and *S. marcescens *permit comprehensive molecular analyses, these two species provide a promising model system for inference of the molecular basis of such highly specific interactions, which are as yet unexplored in invertebrate hosts.

## Background

By definition, parasites have a negative effect on host fitness. Since parasites usually show a shorter generation time than their hosts, they are also able to adapt rapidly to newly arising host genotypes. Both characteristics together select for hosts with efficient counter-adaptations. Subsequently, parasites are favoured if they can circumvent these host countermeasures. Such interactions may result in a co-evolutionary arms race, consisting of repeated cycles of the emergence of new parasite offences and host countermeasures. Hence, parasite-host interactions can lead to extremely rapid evolutionary change [1,2]. As such, they are thought to be responsible for much of the complexity found in the immune system of animals [3]. They are also likely to account for the evolution of diverse genetic mechanisms, which aid in generating fast changes, including sexual reproduction and recombination [4,5]. They may also affect the evolution of other life-history traits, such as reproductive rate, longevity, or competitive ability, which compete for available resources with defence and virulence traits in host and parasite, respectively [6,7].

Co-evolutionary arms races between hosts and parasites (meaning here eukaryotic organisms, bacterial pathogens, and viruses that gain a fitness advantage from infecting and harming a host) are in many cases assumed to be determined by negative frequency dependent selection. In particular, rare parasite and host genotypes should be at an advantage because commonness facilitates evolution of host or parasite counter-adaptations, respectively [4,5]. Such frequency dependent dynamics rely on two important conditions: i) natural genetic variation in both host resistance and parasite virulence, and ii) natural genotype-specific interactions between hosts and parasites [1,4]. Empirical evidence for the presence of both of these prerequisites is still rare, especially for invertebrate hosts [8,9]. They include various associations between snails and trematodes (e.g., *Potamopyrgus antipodarum *versus Microphallus [10] or *Bulinus globosus *versus Schistosoma [11]), the association between the waterflea *Daphnia magna *and its microparasite *Pasteuria ramosa *[8], between *Drosophila melanogaster *and its parasitoid *Asobara tabida *[12], between the bumble bee *Bombus terrestris *and the trypanosome *Crithidia bombi *[13], or between the copepod *Macrocyclops albidus *and the cestode *Schistocephalus solidus *[14]. Clearly, more data is needed to determine the importance of parasite-mediated co-evolutionary arms races in nature.

In this study, we evaluated differences in host resistance and parasite virulence, both defined in a broad sense and reciprocally as the effect of an infection on host condition (i.e. alive, morbid, or dead). In particular, we tested the presence of genetic variation and also the presence of strain- and genotype-specific interactions during the infection of the nematode *Caenorhabditis elegans *(Nematoda: Rhabditidae) by the Gram-negative bacterium *Serratia marcescens *(Enterobacteriaceae). *C. elegans *has recently been established as a model to study parasite-host interactions and in particular the genetics of host defence [15-17]. It is a soil inhabitant found in almost all temperate regions of the world. It seems to be common in decomposing material, where it feeds on diverse microorganisms [18,19]. About 50 natural strains are currently available. These strains are genetically very diverse, even when isolated from populations at a single location [18,19]. They also differ in many life-history traits, including their response towards the potential parasite *Bacillus thuringiensis *[20]. The parasites that *C. elegans *encounters under natural conditions have not yet been unambiguously identified. The ubiquitous soil-dwelling bacteria *Pseudomonas aeruginosa*, *B. thuringiensis *and *S. marcescens *are all likely candidates [17]. For our study, we chose *S. marcescens*, recently adopted as a model to study the genetic basis of virulence [15,21], as a pathogen as it is able to produce a persistent infection and it is likely to benefit from the infection [22], thus behaving as a true parasite of the nematode.

## Results

In our main experiment, we compared the consequences of infection of eight different natural *C. elegans *strains with 5 different *S. marcescens *strains plus one control (heat-killed bacteria of *S. marcescens *Db11, a strain for which the genome sequence is now complete). The *C. elegans *strains were isolated from Münster, in Northwest Germany, and belong to four different microsatellite genotypes [18]. The *S. marcescens *strains originate from different locations around the world. The interaction between the two species was examined with the help of a survival assay, in which the survival of individual worms was monitored in the presence of a defined concentration of bacteria [20]. The survival assay was performed in 96-well plates on five occasions (runs). During each run, all possible bacterial and worm strain combinations were assayed in parallel, resulting in a total of 16 data points per factor combination per run and 80 data points per factor combination in total.

80 out of a total 3840 cases (2.08%) had to be excluded because of errors during automated worm-transfer (either no worm or more than one worm per well), resulting in between 75 and 80 usable data points per combination of worm and bacterial strains. The number of valid cases did not differ significantly among these factor combinations (likelihood ratio test [LRT], *χ*^2 ^= 0.904, *d.f. *= 35, *P *> 0.999). In the control (worms with dead *S. marcescens *Db11), only 12 out of 625 were not found in the category "alive" (1.92%). Of these, 9 were morbid and 3 were dead. The recorded number of live worms per strain did not differ significantly from 100% (LRT, *χ*^2 ^= 0.455, *d.f. *= 7, *P *> 0.999). It also did not differ significantly among the worm strains (LRT, *χ*^2 ^= 0.416, *d.f. *= 7, *P *> 0.999). These results show that the experimental set-up itself does not cause significant levels of dead or morbid worms and that it does not have a different effect on different worm strains.

**Figure 1:**
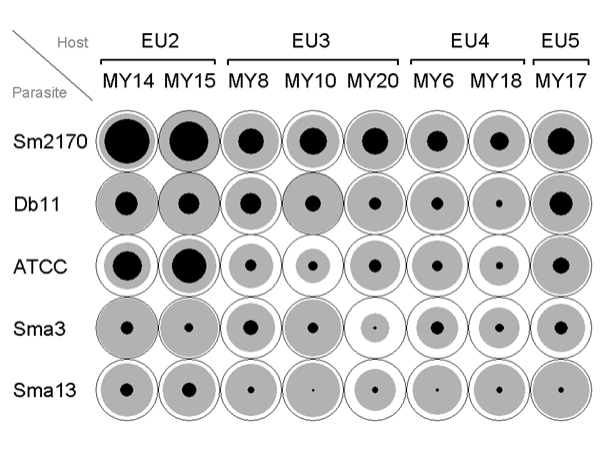
**Treatment response for the different bacterial and worm strain combinations of the main experiment. **The response is expressed as host condition (values for the whole experiment), such that the black area refers to the proportion of dead worms, grey to the proportion of morbid, and white to the proportion of live worms. For *C. elegans*, both strain (bottom line) and genotype (top line) designations are given. For *S. marcescens*, only strain names are listed.

The different *C. elegans *strains show substantial differences as to their ability to survive in the presence of pathogenic *S. marcescens *(Fig. 1). In general, the strains MY6 and MY18 were most resistant, whereas MY14 and MY15 were most susceptible. Moreover, the strains with identical microsatellite genotypes generally produce similar but not identical levels of resistance. This suggests that these strains bear additional genetic differences, which were not resolved by microsatellite genotyping. At the same time, the different *S. marcescens *strains differ considerably in their effect on *C. elegans *(Fig. 1). Here, strain Sm2170 was most virulent, whereas strains Sma3 and Sma13 generally produced the fewest cases of mortality and morbidity. Since *S. marcescens *strains were grown under identical conditions and since some of them are already known to differ in phenotype (e.g. red pigmentation), the observed differences are most likely determined genetically. Most interestingly, the interaction between specific worm and bacterial strains seems to differ across the table. For instance, *C. elegans *strain MY10 is more susceptible to *S. marcescens *strain Sma13 than to ATCC274, whereas the opposite is true for *C. elegans *strain MY20 (Fig. 1). Similarly, host strain MY15 is more susceptible to pathogen strain ATCC274 than to strain Db11, whereas the pattern is reversed for almost all other host strains (Fig. 1).

In general consistency with these observations, ordinal logistic regression (OLR) analysis indicates a significant effect of the factors bacterial strain, worm strain or genotype, the interaction between the two, and also experimental run on the treatment response (Table 1). The two respective models employed are significantly better than models without any predictors (model including worm strain as factor: LRT, *χ*^2 ^= 1285.63, *d.f. *= 199, *P *< 0.0001; model including worm microsatellite genotype as factor: LRT, *χ*^2 ^= 854.55, *d.f. *= 99, *P *< 0.0001). However, they are both significantly worse than the respective saturated models (model including worm strain: LRT, *χ*^2 ^= 491.32, *d.f. *= 199, *P *< 0.0001; model including worm genotype: LRT, *χ*^2 ^= 442.94, *d.f. *= 99, *P *< 0.0001). The latter test examines whether the model employed considers a sufficient number of factors or factor combinations to explain the variation found in the data. The results suggest that the model is not sufficiently complex. We decided against employing more complex models (e.g. consideration of host genotype nested in host strain in a single model), because the response variable is ordinal with only three categories (alive, morbid, dead), such that a larger number of predictor variables in the model would most likely lead to highly increased random error in the regression analysis. Thus, as an alternative, we analysed the data using association tests.

**Table 1:** Ordinal logistic regression analysis of the importance of different factors in the main experiment.

Source	*χ*^2^	*d.f.*	*P*
Consideration of worm strains as a factor			
Bacteria	272.78	4	**< 0.0001**
Worm	188.11	7	**< 0.0001**
Bacteria*Worm	127.15	28	**< 0.0001**
Run [Bacteria, Worm]	835.27	160	**< 0.0001**
Consideration of worm genotypes as a factor			
Bacteria	193.77	4	**< 0.0001**
Worm	169.87	3	**< 0.0001**
Bacteria*Worm	34.21	12	**0.0006**
Run [Bacteria, Worm]	477.14	80	**< 0.0001**

Ordinal logistic regression was based on a model, which contained bacterial strain, worm strain (alternatively worm genotype), the interaction between the two and run nested within both bacterial strain and worm strain/genotype as factors. The importance of different factors was assessed with the likelihood ratio test. Significant probabilities after Dunn-Sidák correction are given in bold.

Two-way associations were analysed with the LRT. The results show a significant effect of either of the different factors on worm condition (Table 2). The relevance of these associations was further examined by taking into account a second predictor variable using the Cochran-Mantel-Haenszel (CMH) test of conditional independence. All previously identified associations remained significant, irrespective of the second predictor variable considered (Table 2). The only exception refers to the case where the factor worm strain was corrected by the factor worm genotype, suggesting that the observed variation among *C. elegans *strains is due to differences in genotypic composition. The remaining results indicate that the significant effect from one of the factors on the treatment response is independent of the significant effect from one of the other factors. This finding is consistent with the presence of an interaction effect from the factors bacterial strain and nematode strain/genotype, as above suggested by OLR.

**Table 2:** Association analysis of the impact of different factors on worm condition in the main experiment .

Factor	Test	*χ*^2^	*d.f.*	*P*
Single factor effects				
Bacteria	LRT	291.05	8	**< 0.0001**
Worm strain	LRT	154.84	14	**< 0.0001**
Worm genotype	LRT	136.29	6	**< 0.0001**
Run	LRT	186.33	8	**< 0.0001**
Factor effects in consideration of one of the others (in brackets)				
Bacteria (Worm strain)	CMH	196.74	4	**< 0.0001**
Bacteria (Worm genotype)	CMH	196.44	4	**< 0.0001**
Bacteria (Run)	CMH	192.08	4	**< 0.0001**
Worm strain (Bacteria)	CMH	146.21	7	**< 0.0001**
Worm strain (Run)	CMH	139.83	7	**< 0.0001**
Worm strain (Worm genotype)	CMH	10.32	7	0.1713
Worm genotype (Bacteria)	CMH	135.50	3	**< 0.0001**
Worm genotype (Run)	CMH	129.34	3	**< 0.0001**
Run (Bacteria)	CMH	52.56	4	**< 0.0001**
Run (Worm strain)	CMH	51.09	4	**< 0.0001**
Run (Worm genotype)	CMH	50.89	4	**< 0.0001**

The associations were assessed with the likelihood ratio test (LRT) or the Cochran-Mantel-Haenszel (CMH) test. Bold probabilities are significant after Dunn-Sidák correction.

In the second experiment, we specifically addressed the presence of an interaction between two bacterial strains (Db11, ATCC274) and four host strains (MY8, MY10, MY14, MY15), the latter belonging to two different host genotypes. For this experiment, all factor combinations were included in each 96-well plate and in one experimental run. Only 7 out of 384 cases had to be excluded for the reasons given above (1.82%), resulting in 46 to 48 data points per factor combination. Again, the number of valid cases did not differ among factor combinations (LRT, *χ*^2 ^= 0.093, *d.f. *= 3, *P *= 0.996). In the control treatment of this experiment, all animals were alive.

**Figure 2:**
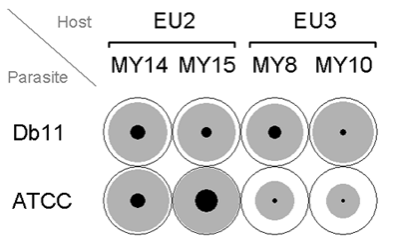
**Treatment response for the different bacterial and worm strain combinations of the second experiment. **The black area denotes the proportion of dead worms, grey the proportion of morbid, and white the proportion of live worms.

The second experiment confirmed the presence of variation in host resistance and pathogen virulence, although the overall level of virulence was lower than in the main experiment (Fig. 2). Subsequent OLR revealed a significant effect from the factor worm strain or worm genotype, and also the interaction between the bacterial strain and either worm strain or genotype. The effect of bacterial strains was significant before Dunn-Sidák adjustment of significance levels (due to multiple testing), but insignificant afterwards (Table 3). For these OLR analyses, the models employed were significantly better than a model without any predictors (model including worm strain as factor: LRT, *χ*^2 ^= 62.29, *d.f. *= 7, *P *< 0.0001; model including worm microsatellite genotype as factor: LRT, *χ*^2 ^= 59.12, *d.f. *= 3, *P *< 0.0001). Moreover, they were not significantly worse than the respective saturated models (model including worm strain: LRT, *χ*^2 ^= 4.62, *d.f. *= 7, *P *= 0.7060; model including worm genotype: LRT, *χ*^2 ^= 1.32, *d.f. *= 3, *P *= 0.7238), suggesting that they contained sufficient details to explain the observed variation.

**Table 3:** Ordinal logistic regression analysis of the importance of different factors in the second experiment.

Source	*χ*^2^	*d.f.*	*P*
Consideration of worm strains as a factor			
Bacteria	4.89	3	0.0270
Worm	33.20	1	**< 0.0001**
Bacteria*Worm	26.89	3	**< 0.0001**
Consideration of worm genotypes as a factor			
Bacteria	4.80	1	0.0284
Worm	31.97	1	**< 0.0001**
Bacteria*Worm	24.50	1	**< 0.0001**

Ordinal logistic regression was based on a model, which contained bacterial strain, worm strain (alternatively worm genotype), and the interaction between the two as factors. The importance of different factors was assessed with the likelihood ratio test. Bold probabilities indicate significance after Dunn-Sidák correction.

Subsequent performance of association tests generally corroborated the OLR analyses: The different predictor variables had a significant effect on the treatment response (LRT analysis in Table 4). With two exceptions, this was still true after correcting for one of the other predictors (CMH tests in Table 4). One of the exceptions refers to the factor bacterial strain, which no longer produced a significant effect if corrected by any of the other factors. This is in agreement with results from the OLR analysis. The other case shows that the factor worm strain becomes insignificant if corrected by worm genotype, which confirms the findings for the main experiment (see above). Consequently, the results clearly demonstrate that there are significant differences among host genotypes and, most importantly, that there are significant strain- or genotype-specific interactions between the two species.

**Table 4:** Association analysis of the impact of different factors on worm condition in the second experiment.

Factor	Test	*χ*^2^	*d.f.*	*P*
Single factor effects				
Bacteria	LRT	10.31	2	**0.0058**
Worm strain	LRT	32.80	6	**< 0.0001**
Worm genotype	LRT	30.46	2	**< 0.0001**
Factor effects in consideration of one of the others (in brackets)				
Bacteria (Worm strain)	CMH	4.34	1	0.0372
Bacteria (Worm genotype)	CMH	4.41	1	0.0358
Worm strain (Bacteria)	CMH	29.21	3	**< 0.0001**
Worm strain (Worm genotype)	CMH	0.93	3	0.8193
Worm genotype (Bacteria)	CMH	28.37	1	**< 0.0001**

The associations were assessed with the likelihood ratio test (LRT) or the Cochran-Mantel-Haenszel (CMH) test. Bold probabilities are significant after Dunn-Sidák correction.

## Discussion

We here provide evidence for the presence of i) genetic differences in resistance among natural *C. elegans *strains, ii) genetic differences in virulence among natural *S. marcescens *strains, and also iii) strain- or genotype-specific interactions between the two. The first of these points is generally consistent with our previous results on the presence of strain-specific differences in resistance of *C. elegans *towards *Bacillus thuringiensis *[20]. However, in the previous study, we compared *C. elegans *strains from different locations across the world, whereas in the present study all strains derive from the same place (the town of Münster in Northwest Germany) [18]. Previous microsatellite genotyping demonstrated that these strains are genetically extremely diverse [18]. Our present results highlight the fact that genetic diversity translates into phenotypic differences in resistance. Importantly, as these differences are present in one population, they could provide the basis for and/or represent the outcome of evolution under natural conditions. These conclusions are restricted to the host *C. elegans*, because the *S. marcescens *strains considered did not come from the same location.

The observed strain- and genotype-specific interactions represent an important precondition for negative frequency dependent selection. As such, they may contribute to the emergence of co-evolutionary arms races [1,4]. The relevance of our results for the association between *C. elegans *and *S. marcescens *in the wild must currently be considered unclear. To date, it is unknown whether the two species indeed co-exist under natural conditions, even though it is strongly suggested by the fact that both – especially *S. marcescens *– are common soil inhabitants [19,23]. If they do co-exist, they clearly show the potential to engage in co-evolutionary interactions. In fact, in this case, the observed specificity may represent a signature of past counter-adaptations. Our results would then also suggest that such highly specific interactions are widespread among invertebrate hosts; they are currently only known in a few arthropods and molluscs (see the background section for examples).

The situation is clearly different if the two species do not share the same natural habitat. In this case, the observed specificity must be the result of independent adaptations of parasite and host strains to other environmental conditions. Pleiotropy of such adaptations should then have produced the specific *C. elegans*-*S. marcescens *interactions as a side effect. For example, the *C. elegans *strains may have adapted differently towards environmental toxins. If the underlying detoxification mechanisms are also employed in the defence against pathogens, then this may result in the observed differences in resistance. Such mechanisms could indeed be of relevance in the interaction with *S. marcescens*, for which at least one toxin (hemolysin ShlA) was previously suggested to contribute to pathogenesis in *C. elegans *[22]. Moreover, such mechanisms may also account for highly specific interactions, even if the two species did co-exist in the wild, underlining the idea that past co-evolutionary events cannot be reliably deduced from the observation of specific interactions without further information (e.g. historical records of co-existence in nature or congruent phylogenies of host and parasite strains).

Whatever its origin, the finding of high specificity in the interaction has further implications. The molecular basis of highly specific resistance is currently unexplored in invertebrate hosts. It could be due to the presence of different alleles of a certain cell surface protein targeted by specific parasite effector molecules. Such cell surface proteins have been suggested to be important for the interaction between *C. elegans *and Bt toxin, the main virulence factor of *B. thuringiensis *[24,25]. As a non-exclusive alternative, specificity may be a consequence of the inducible immune system as recently suggested for the specific interactions between the copepod *M. albidus *and the cestode *S. solidus *[14] or the waterflea *D. magna *and its microparasite *P. ramosa *[26]. The presence of an inducible system was recently demonstrated for *C. elegans *in response to *S. marcescens *[27], the fungus *Drechmeria coniospora *[28], and also the Bt toxin of *B. thuringiensis *[29]. Considering that diverse molecular tools are available for *C. elegans*, this nematode may in the future provide a valuable model system to dissect the molecular basis of specificity in invertebrate-pathogen interactions.

Similarly, the observed highly specific virulence was previously unknown for *S. marcescens*. This bacterium is considered to be an opportunistic pathogen with a broad host range [23]. Hence, it should mainly possess unspecific virulence factors, which are effective against a large number of different taxa. Interestingly, some of the genes previously identified to contribute to pathogenesis in *C. elegans *also mediate virulence in other hosts (*Drosophila melanogaster*; mice), whereas other genes do not [22]. This already indicates some degree of specificity. Our results may now provide the basis for a molecular genetic characterisation of virulence factors that vary in their specific effects against different strains of a single host species. This information may potentially be of great value for understanding pathogenicity of *S. marcescens *in humans, where this bacterium has become a growing health problem, primarily in nosocomial infections [30].

## Conclusions

Based on the analysis of natural isolates of the nematode *C. elegans *and its potential microparasite *S. marcescens*, our study provides evidence for i) genetic variation in host susceptibility and parasite virulence, and also ii) strain- and genotype-specific interactions between the two. These two factors represent an important precondition for frequency dependent selection and as such for the emergence of co-evolutionary arms races. Such highly specific interactions were previously unknown for *C. elegans *or *S. marcescens*. Moreover, they have not as yet been reported for invertebrates other than molluscs and arthropods. At least for *C. elegans*, the observed variation was found among strains from the same population, such that it could indeed be of relevance for evolutionary changes under natural conditions. Taken together, these findings suggest that there is widespread potential for co-evolutionary interactions in animal hosts. Both *C. elegans *and *S. marcescens *represent important model organisms in biological research for which a diversity of manipulative techniques is available. Therefore, the association between these two species may in the future provide a valuable tool for the comprehensive analysis of such highly specific interactions.

## Methods

We compared eight different natural *C. elegans *strains with 5 different natural *S. marcescens *strains plus one control. The *C. elegans *strains were isolated by HS and co-workers in 2002 from Münster, North-West Germany [18]. They are available from the Caenorhabditis Genetics Centre under strain numbers MY6, MY8, MY10, MY14, MY15, MY17, MY18, MY20 [31]. Some of these strains bear different genotypes: strains MY6 and MY18 have genotype EU4; MY8, MY10 and MY20 genotype EU3; MY14 and MY15 genotype EU2; and MY17 genotype EU5 [18]. Maintenance of worms, including feeding, worm transfer, synchronisation of cultures and cryo-preservation followed standard procedures [32]. These *C. elegans *strains had all been cryo-preserved within 5 generations after isolation [18]. They were then thawed only few generations before the start of the experiments to ascertain that they were subjected to selection towards laboratory conditions for the shortest possible time. One generation before the start of the experiment, the worm cultures were always synchronised using NaOH/NaOCl-treatment [32].

The *S. marcescens *strain Db11 was originally isolated by H. Boman [33]; the strain Sm2170 was obtained from T. Watanabe [34]; and strains ATCC274 [35], Sma3, Sma13 from G. Salmond (Cambridge, UK). These strains are known to differ in pigmentation, which is thought to correlate with virulence: Db11, Sma3, and Sma13 have no pigments, whereas ATCC274 and Sm2170 are pigmented [22]. They were also already shown to differ in virulence towards the main *C. elegans *strain N2 when tested on solid agar, whereby virulence varied in the following order (from high to low): Sm2170 > ATCC274 > Db11 > Sma3 = Sma13 [22]. Note, however, that the time-course of infection in liquid medium is much more rapid than on solid medium and that the underlying mechanisms of pathogenesis in the two cases are not identical, at least for Db11 (JJE and E. Pradel, unpublished observations). One day before the start of the experiment, the bacteria were grown in Luria Broth (LB) for about 18 h at 37°C. Their OD was then adjusted to a value of 0.1 by addition of LB. An OD 0.1 corresponds to a cell count of approximately 2 × 10^8 ^per ml. As a control, we used heat-killed bacteria of strain Db11 (incubation at 70°C for 15 min). These dead bacteria were previously shown to have lost their deleterious effects on *C. elegans *[22].

The interaction between *C. elegans *and *S. marcescens *was assessed using a simple survival assay [20]. For this, individual worms were confronted with a defined concentration of the pathogens in NGM solution and their survival checked after 24 h. The experiment was performed in 96-well plates. Each plate always contained the five different *S. marcescens *strains and the control, randomised across the plate. Only one *C. elegans *strain was examined per plate. Eight plates were analysed in parallel, each with one of the eight different *C. elegans *strains. Five runs of this set-up were performed, whereby the order in which strains were analysed during each run was randomised. This set-up resulted in a maximum of 80 data points per bacterial strain – worm strain combination.

For a specific run, 50 μl NGM solution (without any bacteria) were first added to each well of the 96-well plates using a multi-channel pipette. Thereafter, individual worms were transferred to each well using the COPAS automated worm-sorter (Union Biometrica Inc.). For worm-sorting, we used synchronised L4 stage worms. Success of worm transfer was monitored. If no worm or more than one worm was transferred to a particular well, then it was excluded from further analysis. After worm transfer was completed, 50 μl NGM solution with pathogens were added to each well with a multi-channel pipette. These 50 μl contained 45 μl NGM solution and 5 μl of bacteria in LB with an OD of 0.1, resulting in a total of approximately 1 × 10^7 ^bacterial cells per well at the start of the survival assay. This concentration was found in a pilot study to permit detection of differences in survival among worm strains after 24 h. After this time period, the condition of the worms was recorded using the following three categories: i) alive (clearly visible body movements; in some cases only after being touched with a small pipette tip), ii) morbid (touching them with a small pipette tip resulted in retarded, very slow movements), iii) dead (no movements, even after being touched with a small pipette tip).

After completion of the experiment, we re-assessed the interaction between two bacterial strains (Db11 and ATCC274) and four worm strains (MY8, MY10, MY14, MY15; the first two and the last two have identical microsatellite genotypes). The general set-up was the same as above. In this case, a total of four 96-well plates were studied at the same time. In contrast to the above experiment, each 96-well plate contained both the different worm and bacterial strains, randomised across the plate. This set-up results in a maximum of 48 data points per factor combination. In addition to the above, we included two 96-well plates as a control. These contained heat-killed Db11 bacteria and the four worm strains randomised across plates (48 data points per worm strain). After exposure to the pathogens, we confirmed that worms were indeed infected with the bacteria by analysis of some of the animals (*N *= 40) using differential interference contrast microscopy and a fluorescent microscope (DMIRBE, Leica).

The statistical analysis was performed with the program JMP version 5.0 (SAS Institute Inc.). Based on the hierarchical order of the categorical response variable (0, dead; 1, morbid; 2, alive), we used an ordinal logistic regression analysis (OLR) [36,37]. For the main experiment, we included bacterial strain, worm strain, the interaction between the two, and also run nested within both bacterial and worm strain as factors in the model. The whole analysis was repeated using worm microsatellite genotypes instead of worm strain in the model. For the second experiment, which was performed on a single occasion instead of separate runs, a full factorial model was employed, including bacterial strain, worm strain (alternatively, microsatellite genotype), and the interaction between the two as factors.

For the main experiment, the lack of fit test was significant, indicating that the chosen model may not be sufficiently complex to explain the variation in the data (see results section). Therefore, we additionally employed association tests based on the inferred frequency counts for the different factor combinations. We specifically assessed the association between the treatment response (condition of the worms) and either of the following factors: bacterial strain, worm strain, worm microsatellite genotype, and run. The significance of the association was inferred using the likelihood ratio test (LRT) [36,38]. We further used the Cochran-Mantel-Haenszel test (CMH) to assess the conditional independence between the treatment response and one of the above factors in consideration of a second factor from the above list [36]. The response variable was treated as ordinal, while the predictors were treated as nominal (ordinal-nominal conditional association test) [36]. Multiple testing was accounted for by adjusting the significance level using the Dunn-Sidák procedure [38].

## List of abbreviations used

CMH, Cochran-Mantel-Haenszel test; LB, Luria broth; LRT, likelihood ratio test; NGM, nematode growth medium; OD, optical density; OLR, ordinal logistic regression.

## Authors' contributions

HS designed the study, carried out the experiments, analysed the data and wrote the first draft of the manuscript. JJE participated in the design of the study, the interpretation of the data and revised the manuscript.
